# Hybrid Quinoline-Sulfonamide Complexes (M^2+^) Derivatives with Antimicrobial Activity

**DOI:** 10.3390/molecules25122946

**Published:** 2020-06-26

**Authors:** Dumitrela Diaconu, Violeta Mangalagiu, Dorina Amariucai-Mantu, Vasilichia Antoci, Cristian Levente Giuroiu, Ionel I. Mangalagiu

**Affiliations:** 1 Faculty of Chemistry, Alexandru Ioan Cuza University of Iasi, 11 Carol Bvd, 700506 Iasi, Romania; cucu.dumitrela@yahoo.com (D.D.); dorinaiasi@yahoo.com (D.A.-M.); vasilichia2004@yahoo.com (V.A.); 2 Institute of Interdisciplinary Research—CERNESIM Center, Alexandru Ioan Cuza University of Iasi, 11 Carol Bvd, 700506 Iasi, Romania; 3 Endodontics, Faculty of Dental Medicine, Grigore T. Popa University of Medicine and Pharmacy, 16 Universităţii Street, 700115 Iasi, Romania

**Keywords:** hybrid quinoline-sulfonamide complexes, small-molecule drugs, antimicrobial activity

## Abstract

Two new series of hybrid quinoline-sulfonamide complexes (M^2+^: Zn^2+^, Cu^2+^, Co^2+^ and Cd^2+^) derivatives (**QSC**) were designed, synthesized and tested for their antimicrobial activity. The synthesis is straightforward and efficient, involving two steps: acylation of aminoquinoline followed by complexation with metal acetate (Cu^2+^, Co^2+^ and Cd^2+^) or chloride (Zn^2+^). The synthesized **QSC** compounds were characterized by FTIR and NMR spectroscopy and by X-ray diffraction on single crystal. The **QSC** compounds were preliminary screened for their antibacterial and antifungal activity and the obtained results are very promising. In this respect, the hybrid *N*-(quinolin-8-yl)-4-chloro-benzenesulfonamide cadmium (II), considered as leading structure for further studies, has an excellent antibacterial activity against *Staphylococcus aureus ATCC25923* (with a diameters of inhibition zones of 21 mm and a minimum inhibitory concentration (MIC) of 19.04 × 10^−5^ mg/mL), a very good antibacterial activity against *Escherichia coli ATCC25922* (with a diameters of inhibition zones of 19 mm and a MIC of 609 × 10^−5^ mg/mL), and again an excellent antifungal activity against *Candida albicans ATCC10231* (with a diameters of inhibition zones of 25 mm and a MIC of 19.04 × 10^−5^ mg/mL).

## 1. Introduction

Despite of the significant advances in the antimicrobial therapy accomplished in the last few decades, infectious diseases caused by microorganisms (bacteria, fungus, viruses, *Mycobacterium tuberculosis*, etc.) represent seriously threaten of modern medicine and global public health. Drug resistance, multi-drug resistance and extensively-drug-resistance are the leading cause of these drawbacks, but some other causes could be also taken into consideration [1,2,3]. Quinoline based compounds are small molecules of huge importance from pharmacological point of view, having a wide range of biological activities such as antiplasmodial and antimalarial, antibacterial, antifungal, antitubercular, anti-HIV, antiviral (including against COVID-19), etc. [4,5,6,7,8,9,10,11,12,13,14,15,16]. A special class of quinoline derivatives which pay a particularly attention on scientific community, are quinoline-sulfonamide complexes (**QSC**), studied especially for their fotoluminiscent (mostly fluorescent) properties [17,18,19,20,21,22,23]. As far for biological activity, these compounds were tested mostly as antiprotozoals [24,25] and very few data was found for their antibacterial and antifungal activity [26]. The antibacterial activity of azaheterocycles sulfonamides is well known [1,2].

Encouraged by our recent results in the field of (di)azine with antimicrobial activity [11,15,27,28,29,30,31,32,33,34,35,36,37,38,39,40], we report here the design, synthesis, antibacterial and antifungal evaluation of some newly hybrid quinoline-sulfonamide complexes.

## 2. Results and Discussion

### 2.1. Design and Chemistry

**Scheme 1:**
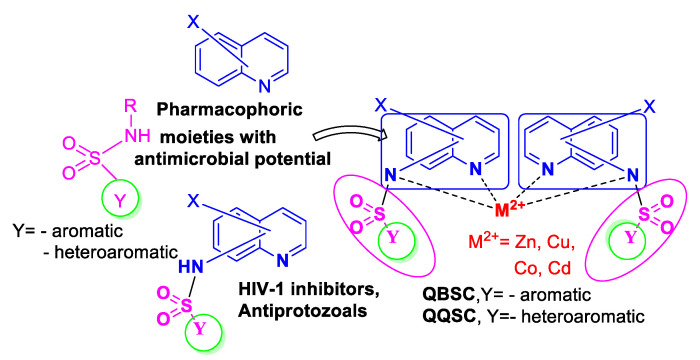
Design in the class of hybrid quinoline-sulfonamide complexes derivatives.

Having in view the biological potential of quinoline and sulfonamide scaffolds (especially antimicrobial) [9,10,11,12,13,14,15], as well as those one of quinoline-sulfonamide combined scaffold (especially anti-HIV-1) [16], we decide to combine the pharmacophoric properties of these core scaffolds with the complementary biological properties of counter cation M^2+^ (M^2+^: Zn^2+^, Cu^2+^, Co^2+^ and Cd^2+^), with the final goal of obtaining better biological activity and better pharmacokinetic properties for our compounds. In this respect, we design two new classes of hybrid quinoline-sulfonamide complexes, namely *N*-(quinolin-8-yl)-4-R-*benzene* sulfonamide metal (II) (**QBSC**) and *N*-(quinolin-8-yl)-quinoline -8-sulfonamide metal (II) (**QQSC**), Scheme 1.

**Scheme 2:**
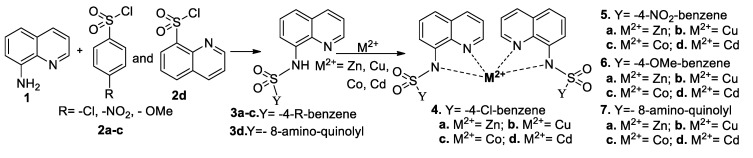
Reaction pathways to obtain hybrid quinoline-sulfonamide complexes derivatives.

As in related cases [21,22,23,24], the general route to obtain the desired compounds involves a simple and efficient two-step procedure. The first step consist in the acylation of (3, 4 or 8)amino-quinoline with variously 4-R-benzenesulfonyl chlorides (R = Cl, NO_2_, OMe) and quinolylsulfonyl chlorides, when the corresponding ligands quinoline-sulfonamide type **3** are obtained. The second step consist in the complexation of ligands **3** with metal acetate (Cu^2+^, Co^2+^, Cd^2+^) or chloride (Zn^2+^). In this way, we obtained the two classes of compounds desired: **QBSC** type **4**–**6** and **QQSC** type **7**. The procedure is depicted in Scheme 2 for the complexes derived from 8-aminoquinoline.

The structures of **QSC** compounds were proved by elemental and spectral analysis (FT-IR, ^1^H-NMR, ^13^C{^1^H}-NMR, and two-dimensional experiments 2D-COSY, HMQC, HMBC), and single crystal X-ray structure determination. If we consider the M^2+^[*N*-(quinolin-8-yl)-4-chloro-benzenesulfonamide]_2_ derivatives **QBSC 4a**–**d** as representative for the hybrid **QSC**, the spectral analysis reveal strong evidence for the proposed structures.

**Figure 1:**
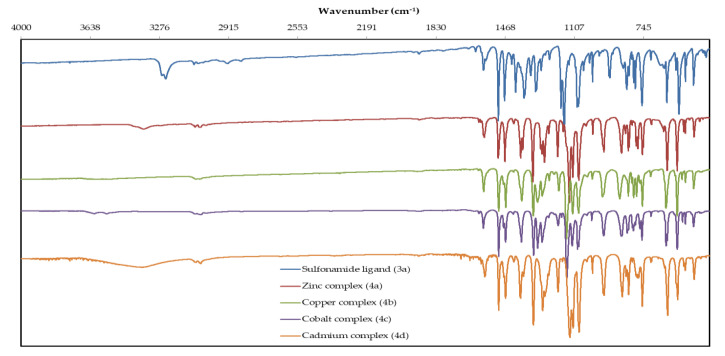
FT-IR spectra of sulfonamide ligand 3a, zinc (II) complex (4a), copper (II) complex (4b), cobalt (II) complex (4c) and cadmium (II) complex (4d).

In the FT-IR spectra of sulfonamide ligand **3a** and its complexes **QBSC 4a**–**d** (Figure 1), the most representative bands, together with their assignments, are given in Table 1.

The band from 3267 cm^−1^ (medium intensity), which is corresponding to the N–H stretching vibration in the free ligand, is missing in the spectra of the complexes, confirming deprotonation of the nitrogen atom of the sulfonamide and its coordination to the metal ion. The bands corresponding to the asymmetric and symmetric stretching vibration of the sulfonyl group in ligand appear at 1372 cm^−1^ respectively at 1177 cm^−1^, while in spectra of the complexes are shifted with ≈20 cm^−1^ to higher wave numbers respectively ≈40 cm^−1^ to lower wave numbers; this is because in the free ligand a larger double bond character of the SO bonds could be incriminated. The band due to the sulfonyl group is split in the spectra of the complexes, because of the different spatial orientation of these groups, as the X-ray show. We also notice a similar shift with ≈20 cm^−1^ to higher wave numbers for the S–N stretching vibration in the spectra of the complexes, probably because the N atom is involved in the bonding to the metal atom. The remaining bands appear at wave numbers in accordance with the proposed structures both in the free ligand and complexes.

**Figure 2:**
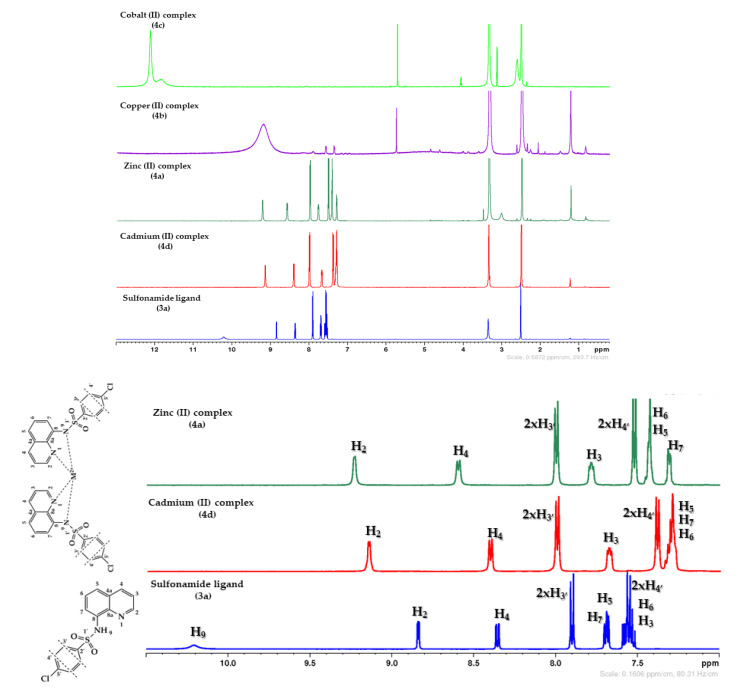
^1^H-NMR spectra for sulfonamide ligand (**3a**) and its complexes (**4a**–**d**). At the bottom part of the image is represented a detail of aromatic zone (7–11 ppm).

The ^1^H-NMR and ^13^C{1H}-NMR spectra of the sulfonamide ligand and its complexes shows characteristic chemical shifts that are in agreement to the proposed structure. The most significant signal in the ^1^H-NMR spectra (Figure 2), corresponding of the hydrogen atom from sulfonamide group **H_9_** and, appear in the free ligand at a chemical shift of 10.20 ppm. This signal is missing in the spectra of **QBSC** complexes, which is a solid prove for the complexation with the metal ion. We could also notice that the signals of hydrogen atoms near the metal ion **H_2_, H_3_, H_4_** (from pyridine ring), are deshielded in **QBSC** complexes with about 0.30 ppm due to the powerful deshielding effect of the metal ion, a electron density transfer from the ligand to the metal ion being responsible for this.

**Figure 3:**
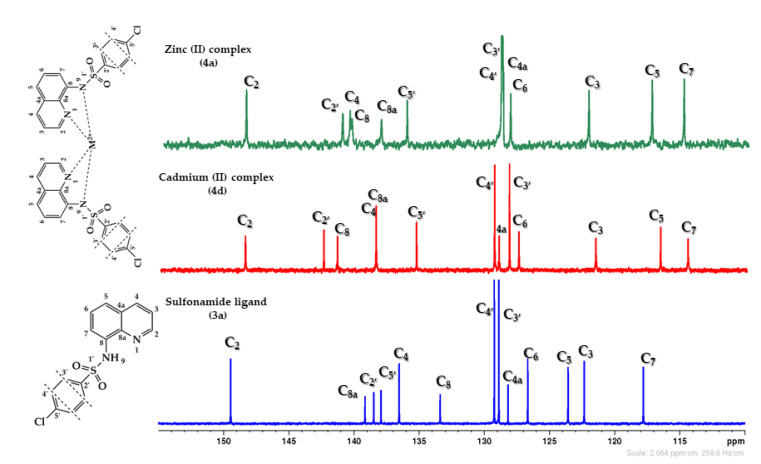
^13^C{^1^H}-NMR spectra detail (110–160 ppm) for sulfonamide ligand (**3a**), Zn (II) and Cd (II) complexes.

Similar considerations could be done for ^13^C{^1^H}-NMR spectra, Figure 3. The most relevant signals in the ^13^C{^1^H}-NMR spectra, correspond to the carbon atoms **C_2′_**, **C_8_**, **C_5_** and **C_7_**. In **QBSC** complexes, the **C_2′_** carbons (*ipso*-sulfonamide) appear to a chemical shift of about 142 ppm due to the powerful deshielding effect of sulfonamide group and the metal ion, while **C_8_** (carbon from attached benzene ring of quinoline) appear to a chemical shift of about 141 ppm, the same reasons being incriminated. Comparative with the free ligand, in **QBSC** complexes, these carbons are deshielded with about 8 ppm for **C_8_**, respectively, with about 4 ppm for **C_2′_,** due to the powerful deshielding effect of the metal ion. The most shielded carbons are **C_5_** and **C_7_**. In **QBSC** complexes, these carbons appear to a chemical shift of about 117 ppm for **C_5_**, respectively, 115 ppm for **C_7_**, being shielded comparative with the free ligand with about 20 ppm for **C_5_** respectively with about 2 ppm for **C_7_.** As far for the NMR spectra of Cu^2+^ and Co^2+^ complexes, the spectra are broad because of their different magnetic properties (paramagnetic).

**Figure 4:**
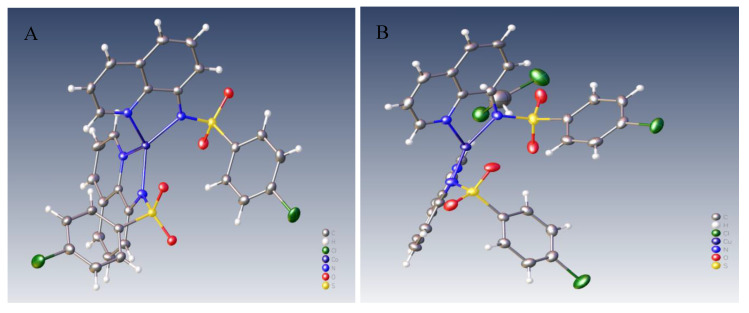
(**A**) Molecular structure of [Co(*N*-(quinoline-8-yl)-4-chloro-benzenesulfonamide)_2_] **4c** complex. (**B**) Molecular structure of [Cu(*N*-(quinoline-8-yl)-4-chloro-benzenesulfonamide)_2_] **4b** complex.

In the case of compounds **4b** and **4c,** the structure of compounds was assigned unambiguously by single-crystal X-ray investigation (for **4a** and **4d**, we did not obtained yet proper crystals), Figure 4A,B.

The structure of **QBSC** cobalt complex **4c** was resolved by direct methods and refined in the monoclinic P_21_/n space group type. Molecular information’s shows a tetrahedral coordination of cobalt with the bidentate ligand trough Co-Nquinoline and Co-Nsulfonamide bonds. The Co-Nquinoline bond lengths (2.038 and 2.029 Å) are slightly larger than Co-Nsulfonamide bonds (1.970 and 1.983 Å) but in an acceptable range as reported by other papers for similar complexes. The structure of **QBSC** copper complex **4b** was resolved by direct methods and refined in the monoclinic I_2_/a space group type. Molecular information’s shows a tetrahedral coordination of copper with the bidentate ligand trough Cu-Nquinoline and Cu-Nsulfonamide bonds. The Cu-Nquinoline bond lengths (1.980 and 1.991 Å) are slightly larger than Cu-Nsulfonamide bonds (1.960 and 1.944 Å) but in an acceptable range as reported by other papers for similar complexes. Copies of NMR spectra of ligand and **QBSC** complexes and checkCIF files for X-ray data of **QBSC** complex **4b** and **4c** are presented to Supplementary Materials.

### 2.2. Antimicrobial Assay

**Figure 5:**
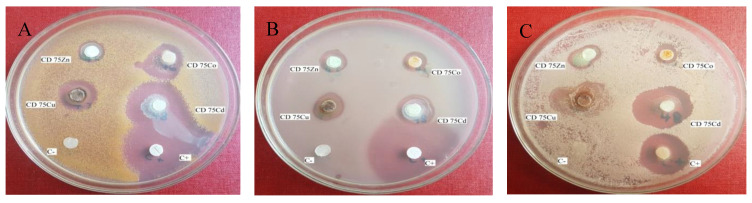
(**A**) The antibacterial activity for **QBSC** compounds **4a** (CD 75Zn), **4b** (CD 75Cu), **4c** (CD 75Co) and **4d** (CD 75Cd) against *S. aureus* (C+: positive control; C−: negative control). (**B**) The antibacterial activity for **QBSC** compounds **4a** (CD 75Zn), **4b** (CD 75Cu), **4c** (CD 75Co) and **4d** (CD 75Cd) against *E. coli* (C+: positive control; C−: negative control). (**C**) The antifungal activity for **QBSC** compounds **4a** (CD 75Zn), **4b** (CD 75Cu), **4c** (CD 75Co) and **4d** (CD 75Cd) against *C. albicans* (C+: positive control; C−: negative control).

The in vitro antimicrobial activity of ligand **3** and **QSC** compounds **4a**–**d** was determined by the Kirby–Bauer disk diffusion method [41] using nutrient agar medium (Mueller Hinton agar for antibacterial tests and Sabouraud agar for antifungal tests). The antibacterial activity was evaluated against two strains bacteria (Gram-positive *Staphylococcus aureus ATCC 25923* and Gram-negative *Escherichia coli ATCC 25922*) and the antifungal activity against fungus *Candida albicans ATCC 10231*. As positive control (C+) was used, Penicillin 10 IU for *Staphylococcus aureus*, Carbenicillin 100 µg/mL for *Escherichia coli* and Nystatin 500,000 IU for *Candida albicans*; the negative control (C−) consist in sterile filter paper disks with no antimicrobial compounds. The more susceptible were the germs, the larger the diameter (mm) of the inhibition zones is. The obtained results are expressed as diameters of inhibition zones (mm) and, for ligand **3,** and **QBSC 4a**–**d** compounds, are presented in Table 2 and Figure 5A–C.

From the data presented in Table 2 and Figure 5A–C, we may notice that some of our **QBSC** compounds demonstrated to be effective against the tested strain. Against bacterial strain *Staphylococcus aureus* one compound **QBSC 4d** is very active (having a diameter of inhibition zone of 21 mm) and three others compounds are active: **QBSC 4c**, **QBSC 4b** and the ligand **3a** (with the diameter of inhibition zone of 17, 16 and 18 mm). Against bacteria *Escherichia coli* one compound **QBSC 4d** is active (19 mm) while against fungus *Candida albicans* two compounds are very active: **QBSC 4d** and the ligand **3a** (25 respectively 26.5 mm).

The compounds which exhibited significant antimicrobial activity were later tested using the standardized broth microdilution assay procedure to determine the minimum inhibitory concentration (MIC) of the compounds under investigation against the reference microorganisms [42]. The resulted MIC value is defined as the lowest concentration of the antimicrobial agent under investigation, which prevents visible growth of the tested microorganism. The obtained results are listed in Table 3.

As we may notice from Table 3, Cd complex (**QBSC 4d**) is active to a very low concentration, having a MIC of 19.04 × 10^−5^ mg/mL in the case of *Staphylococcus aureus* and *Candida albicans*, respectively 609 × 10^−5^ mg/mL in the case of *Escherichia coli*. Significant results was obtained also for Cu complex (**QBSC 4b**) which have MIC value of 4875 × 10^−5^ mg/mL in the case of bacterial streams *Staphylococcus aureus* and *Escherichia coli*.

The diameter of inhibition zone and MIC data reveal that our **QBSC** compounds (except the Zn complexes) have a quasi-nonselective activity against bacteria *Staphylococcus aureus*. Against *Escherichia coli* only Cd complex (**QBSC 4d**) have a significant activity, while against fungus *Candida albicans* Cd complex (**QBSC 4d**) and quinoline sulfonamide ligand (**3a**) present a strong activity. The above presented results also reveal a clear influence of the metal cation, the Cd complex (**QBSC 4d**) being far away the most active tested compound, having a quasi-nonselective antibacterial and antifungal activity. The activity of cadmium complexes are higher the most probable because of a better synergism ligand cadmium.

As to the mechanism of action of our **QBSC** compounds, we may presume that they could act as carbonic anhydrase inhibitors, as the literature describe for related compounds [26,43].

## 3. Experimental

### 3.1. Chemistry

All the reagents and solvents were purchased from commercial sources and used without further purification. Melting points were recorded on a Electrothermal MEL-TEMP II (Barnstead International, Dubuque, IA, USA) apparatus in open capillary tubes and are uncorrected. Analytical thin-layer chromatography (TLC) was performed with commercial Merck silica gel 60 F_254_ plates and visualized with UV light (λ_max_ = 254 or 365 nm). The NMR spectra were recorded on a Bruker Avance III 500 MHz spectrometer (Bruker Vienna, Austria) operating at 500 MHz for ^1^H and 125 MHz for ^13^C. Chemical shifts were reported in delta (δ) units, part per million (ppm) and coupling constants (*J*) in Hz. The following abbreviations were used to designate chemical shift multiplicities: s = singlet, d = doublet, ad = apparent doublet, t = triplet, q = quartet, aq = apparent quartet, m = multiplet. Infrared (IR) data were recorded as films on potassium bromide (KBr) pellets on a FT-IR VERTEX 70 Bruker spectrophotometer. In order to determine the structure of complexes, a crystal of each complex was selected, mounted on a hair thread and inserted into the four-circle SuperNova single crystal diffraction instrument. Data acquisition was made at 100 °K (−173.15 °C) using CuKa radiation. A number of 5608 reflections from a total of 33212 acquired in 2.8345 < θ < 70.8363 range were used for refinement and structure solution.

Compound **3a**, which have already been reported in literature, showed spectral data in agreement to the reported data [16,17,20].

#### 3.1.1. General Procedure for the Synthesis of Sulfonamide Ligand **3a** and its Complexes **4a**–**d**

##### Synthesis of the Ligand

*4-chloro-N-(quinolin-8-yl)benzenesulfonamide* (**3a**), 1 mmol of 8-aminoquinoline was dissolved in minimum volume of dichloromethane CH_2_Cl_2_, then was added to a stirred solution of 4-chlorobenzenesulfonyl chloride (1.1 mmol) and pyridine. The crude product was washed with HCl (1 M), then with a solution of saturated NaHCO_3_. The organic extract was dried on Na_2_SO_4_ and the solvent was removed in vacuum. The solid obtained was purified on column chromatography (CH_2_Cl_2_/AcOEt = 70/30) giving the white solid 4-chloro-*N*-(quinolin-8-yl)benzenesulfonamide.

##### Synthesis of the Complexes

The complexes were prepared by direct reaction between the sulfonamide ligand and Zn(II), Cu(II), Co(II) and Cd(II) salts.

*[Zn(N-(quinoline-8-yl)-4-chloro-benzenesulfonamide)2]* (**4a**), 2 mmol of sulfonamide ligand **3a** were dissolved in 70 mL MeOH and 2 mL NH_4_OH were added. 1 mmol of ZnCl_2_ dissolved in 50 mL methanol were added dropwise while solution was magnetically stirred. When addition is completed, a yellow precipitate is formed, which is separated by filtration. Addition of a base for deprotonating the nitrogen from amine was necessary to prepare the zinc complex. [21]

*[Cu(N-(quinoline-8-yl)-4-chloro-benzenesulfonamide)_2_],[Co(N-(quinoline-8-yl)-4-chloro-benzenesulfonamide)_2_]*, *[Cd(N-(quinoline-8-yl)-4-chloro-benzenesulfonamide)_2_]* (**4b**–**d**), on a methanolic solution of metal (Cu, Co and Cd) (II) acetate (1 mmol) was added dropwise 80 mL of a solution containing 2 mmol of sulfonamide-ligand **3a**. After overnight stirring at room temperature, crystals were formed and they were separated by filtration [23].

*4-chloro-N-(quinolin-8-yl)benzenesulfonamide* (**3a),** White solid; yield: 87%; mp 129–130 °C; IR (KBr), ν_max_ 3267, 1584, 1506, 1372, 1177, 1094, 927, 755, 624, 562 cm^−1^; ^1^H NMR (500 MHz, DMSO-*d_6_*) δ 7.54 (4H, m, 2 × H-4′, H-6, H-3), 7.68 (2H, m, H-7, H-5), 7.89 (2H, d, *J* = 8.5 Hz, 2 × H-3′), 8.35 (1H, dd, *J* = 8.5 Hz, *J* = 1.5 Hz, H-4), 8.84 (1H, dd, *J* = 4.5 Hz, *J* = 1.5 Hz, H-2), 10.20 (1H, s, H-9); ^13^C{1H} NMR (125 MHz, DMSO-*d_6_*) δ 117.79 (C-7), 122.33 (C-3), 123.57 (C-5), 126.66 (C-6), 128.17 (C-4a), 128.88 (2 × C-3′), 129.23 (2 × C-4′), 133.38 (C-8), 136.52 (C-4), 137.91 (C-5′), 138.47 (C-2′), 139.14 (C-8a), 149.45 (C-2); Anal. Calcd. for C_15_H_11_ClN_2_O_2_S C, 56.52; H, 3.48; N, 8.79. Found C, 56.47; H, 3.51; N, 8.74.

*[Zn(N-(quinoline-8-yl)-4-chloro-benzenesulfonamide)_2_]* (**4a**), Yellowish green crystals; yield 92%; IR (KBr), ν_max_ 3390, 1583, 1507, 1471, 1389, 1196, 1138, 954, 753, 624, 572 cm^−1^; ^1^H NMR (500 MHz, DMSO-*d_6_*) δ 7.30 (2H, dd, *J* = 6.0 Hz, *J* = 2.5 Hz, 2 × H-7), 7.43 (4H, ad, *J* = 6.0 Hz, 2 × H-6, 2 × H-5), 7.52 (4H, d, *J* = 8.5 Hz, 4 × H-4′), 7.78 (2H, aq, *J* = 6.5 Hz, 2 × H-3), 8.00 (4H, d, *J* = 8.5 Hz, 4 × H-3′), 8.59 (2H, d, *J* = 7.5 Hz, 2 × H-4), 9.23 (2H, ad, *J* = 3.0 Hz, 2 × H-2); ^13^C{1H} NMR (125 MHz, DMSO-*d6*) δ 115.07 (2 × C-7), 117.52 (2 × C-5), 122.38 (2 × C-3), 128.38 (2 × C-6), 128.94 (2 × C-4a), 129.01 (4 × C-3′), 129.07 (4 × C-4′), 136.32 (2 × C-5′), 138.30 (2 × C-8a), 140.55 (2 × C-8), 140.70 (2 × C-4), 141.26 (2 × C-2′), 148.65 (2 × C-2); Anal. Calcd. for C_30_H_20_C_l2_N_4_O_4_S_2_Zn C, 51.41; H, 2.88; N, 7.99. Found C, 51.47; H, 2.81, N 8.04.

*[Cu(N-(quinoline-8-yl)-4-chloro-benzenesulfonamide)_2_]* (**4b**), Dark brown crystals; yield 85%; IR (KBr), ν_max_ 1583, 1507, 1467, 1385, 1155, 954, 753, 624, 572 cm^−1^; Anal. Calcd. for C_30_H_20_Cl_2_CuN_4_O_4_S_2_ C, 51.54; H, 2.88; N, 8.01. Found C, 51.59; H, 2.80, N 8.06.

*[Co(N-(quinoline-8-yl)-4-chloro-benzenesulfonamide)_2_]* (**4c**), Brick-red crystals; yield 87%; IR (KBr), ν_max_ 1584, 1502, 1468, 1386, 1195, 1151, 961, 755, 624, 572 cm^−1^; Anal. Calcd. for C_30_H_20_Cl_2_CoN_4_O_4_S_2_ C, 51.89; H, 2.90; N, 8.07. Found C, 51.94; H, 2.86, N 8.11.

*[Cd(N-(quinoline-8-yl)-4-chloro-benzenesulfonamide)2]* (**4d**), Beige crystals; yield 79%; IR (KBr), ν_max_ 1578, 1503, 1468, 1323, 1275, 1136, 957, 859, 753, 621, 568 cm^−1^; ^1^H NMR (500 MHz, DMSO-*d_6_*) δ 7.30 (6H, m, 2 × H-7, 2 × H-6, 2 × H-5), 7.38 (4H, d, *J* = 8.5 Hz, 4 × H-4′), 7.78 (2H, aq, *J* = 8.0 Hz, 2 × H-3), 8.00 (4H, d, *J* = 8.0 Hz, 4 × H-3′), 8.40 (2H, d, *J* = 8.0 Hz, 2 × H-4), 9.15 (2H, ad, *J* = 4.5 Hz, 2 × H-2); ^13^C{1H} NMR, (125 MHz, DMSO-*d6*) δ 114.53(2 × C-7), 116.63 (2 × C-5), 121.61 (2 × C-3), 127.51 (2 × C-6), 128.24 (4 × C-3′), 129.04 (2 × C-4a), 129.38 (4 × C-4′), 135.57 (2 × C-5′), 138.46 (2 × C-8a, 2 × C-4), 141.43 (2 × C-8), 142.48 (2 × C-2′), 148.50 (2 × C-2); Anal. Calcd. for C_30_H_20_CdCl_2_N_4_O_4_S_2_ C, 48.18; H, 2.70; N, 7.49. Found C, 48.10; H, 2.78, N 7.42.

### 3.2. Antimicrobial Assay

#### 3.2.1. Disk-Diffusion Method

For inoculum preparation, reference microbial cultures of bacteria (*Staphylococcus aureus ATCC 25923, Escherichia coli ATCC 25922*) and fungi (*Candida albicans ATCC 10231*) were employed. A number of approximately 5 colonies from each type of culture were used to inoculate 10 mL of Mueller Hinton (MH) agar (for antibacterial tests) and Sabouraud agar (for antifungal tests). Using a Beckman Coulter DU 730 spectrophotometer (λ = 600 nm), the turbidity of the inoculum was adjusted to a 0.5 McFarland standard (1–2 × 10^8^ CFU/mL for bacteria and 1–5 × 10^6^ CFU/mL for *Candida*), and the inoculum was transferred, in a 1 mL volume, onto the surface of the growth media specific for bacteria (MH) and fungi (Sabouraud). Once the inoculum was absorbed, sterile paper disks of approximately 6 mm in diameter and impregnated with 10 µL of antibacterial compound (dissolved in DMSO 3%) were placed on the surface of the culture media; for all the tested compounds, the concentration used was 25 mg/mL. Following incubation at the optimal temperatures for bacteria and fungi, of 37 °C and 28 °C, respectively, for 24 h (bacteria) and 72 h (fungi), the diameters of the inhibition zones were measured using a ruler. The controls were prepared in the same growth conditions (i.e., C+: sterile filter paper disks impregnated with antibiotics inducing sensitivity in the organisms under investigation, namely Penicillin 10 IU for *Staphylococcus aureus*, Carbenicillin 100 µg/mL for *Escherichia coli* and Nystatin 500,000 IU for *Candida albicans*, and C−: sterile filter paper disks with no antimicrobial compounds).

#### 3.2.2. Broth Microdilution Method

The working technique involves the use of a 96-well microtiter plate (microdilution). In each well of the plate, 80 µL of growth medium MH, 10 µL of microbial inoculum (*Staphylococcus aureus ATCC 25923, Escherichia coli ATCC 25922, or Candida albicans ATCC 10231*) prepared in the same manner as in the diffusion test (i.e., by diluting the standardized microbial suspension adjusted to a 0.5 McFarland standard), and 100 µL of antimicrobial substance to be tested were transferred by pipetting, in different concentrations. To this purpose, double dilutions of the antimicrobial agent were made in the DMSO 3%, starting with the 25 mg/mL dilution (e.g., 12.5 mg/mL, 6.25 mg/mL, 3.12 mg/mL, 1.56 mg/mL, 0.78 mg/mL and so on). For each tested microorganism, a positive control C+ (containing 80 µL of MH growth medium and 10 µL of antimicrobial compound) and a negative one C- (containing 80 µL of MH growth medium and 10 µL of diluted microbial culture) were prepared. Following the incubation of the microplates at 37 °C for 24 h (for *Staphylococcus aureus ATCC 25923* and *Escherichia coli ATCC 25922*) and at 28 °C for 72 h (for *Candida albicans ATCC 10231*), 10 µL of resazurin were added in each well. The samples were incubated once again at the temperature optimal for each microorganism for one hour. The color of the indicator turned from purple to pink. Resazurin is a colorimetric indicator for cell viability widely applied for monitoring cell proliferation. The redox dye, resazurin, enters the cytosol in the oxidized form (purple–blue) and is converted to the reduced form, resorufin (pink).

## 4. Conclusions

Two new series of hybrid quinoline-sulfonamide complexes (M^2+^: Zn^2+^, Cu^2+^, Co^2+^ and Cd^2+^) derivatives were designed, synthesized and tested for their antimicrobial activity. The synthesis is straight and efficient, involving two steps: acylation of aminoquinoline followed by complexation with metal acetate (Cu^2+^, Co^2+^ and Cd^2+^) or chloride (Zn^2+^). The synthesized compounds were characterized by FTIR, NMR spectroscopy and by X-ray diffraction on single crystal. The Co (II) complex crystallize in the monoclinic P2_1_/n space group type, with a tetrahedral coordination of cobalt with the bidentate ligand trough Co-N_quinoline_ and Co-N_sulfonamide_ bonds. The **QSC** compounds were preliminary in vitro screened for their antibacterial and antifungal activity and the obtained results are very promising. Against bacterial strain *Staphylococcus aureus* one compound have an excellent antibacterial activity **QBSC 4d** (Φ = 21 mm, MIC = 19.04 × 10^−5^ mg/mL) and three others compounds are active: **QBSC 4c**, **QBSC 4b** and the quinoline sulfonamide ligand **3a** (Φ of 17, 16 and 18 mm). Against bacteria *Escherichia coli* only one compound **QBSC 4d** present activity (19 mm, MIC of 609 × 10^−5^ mg/mL). Against fungus *Candida albicans* again the **QBSC 4d** complex have an excellent antibacterial activity (Φ = 25 mm, MIC = 9.04 × 10^−5^ mg/mL) and also the ligand **3a** have an excellent antibacterial activity (Φ = 26.5 mm) but to a relatively high concentration (MIC = 12.5 mg/mL). These results reveal a clear influence of the metal cation, the Cd complex (**QBSC 4d**) being far away the most active tested compound, having a quasi-nonselective antibacterial and antifungal activity. The activity of cadmium complexes are higher the most probable because of a better synergism ligand cadmium.

## Figures and Tables

**Table 1:** Representative bands of sulfonamide ligand **3a** and its complexes with divalent ions Zn^2+^, Cu^2+^, Co^2+^ and Cd^2+^.

ν (cm^−1^)	Sulfonamide Ligand (3a)	Zinc Complex (4a)	Copper Complex (4b)	Cobalt Complex (4c)	Cadmium Complex (4d)
ν_N-H_	3267 (m)	-	-	-	-
ν_asSO2_	1372 (s-m)	1389 (s-m)	1385 (s-m)	1386 (s-m)	1392 (s-m)
ν_symSO2_	1177 (s)	1138 (s)	1155 (s)	1151 (s)	1136 (s)
ν_C-N_	1584 (m)	1583 (m)	1583 (m)	1584 (m)	1578 (m)
ν_S-N_	927 (m)	954 (m)	954 (m)	961 (m)	957 (m)
ν_C-S_	624 (s)	624 (s)	629 (s)	624 (s)	621 (s)

ν—stretching; s—strong; m—medium; w—weak; as—asymmetric; sym—symmetric.

**Table 2:** The antibacterial and antifungal activity for ligand **3a** and **QBSC 4a**–**d** compounds.

Strain		Diameter of	Inhibition Zone		
	^a^ 3a	^a^ 4a (Zn)	^a^ 4b (Cu)	^a^ 4c (Co)	^a^ 4d (Cd)
*S. aureus ATCC 25923*	18 ± 1.73	11 ± 1.73	16 ± 1	17 ± 2	**21**± 2
*E. coli ATCC 25922*	0	12 ± 2	14.5 ± 2	10.7 ± 2	19 ± 1.73
*C. albicans ATCC 10231*	**26.5**± 1.80	12 ± 2	0	11 ± 1	**25**± 1.15

All values represented in the table are average of results of five separately conducted experiments. Underline means active and bold and underline means very active. ^a^ Diameter of inhibition zone (mm), ^a^ X ± SD, mean of five measurements ± standard deviation.

**Table 3:** The minimum inhibitory concentration (MIC) for ligand **3a** and **QBSC 4a**–**d** compounds (mg/mL).

Strain	MIC (mg/mL)				
	3a	4a (Zn)	4b (Cu)	4c (Co)	4d (Cd)
*S. aureus ATCC 25923*	12.5	25	0.04875	12.5	0.00019
*E. coli ATCC 25922*	-	25	0.04875	12.5	0.00609
*C. albicans ATCC 10231*	12.5	12.5	-	12.5	0.00019

## Supplementary Materials

The following are available online: ^1^H- and ^13^C-NMR of compounds **3a**, **4a**, **4d** (pages 1–4), chechCIF reports for compounds **4b** and **4c** (pages 5–11).

## References

[1] Brunton L., Knollmann B., Hilal-Dandan R. (2013). Goodman & Gilman’s the Pharmacological Basis of Therapeutics.

[2] Silverman R.B., Holladay M.W. (2014). The Organic Chemistry of Drug Design and Drug Action.

[3] WHO Global Strategy for Containment of Antimicrobial Resistance. https://www.who.int/drugresistance/WHO_Global_Strategy_English.pdf.

[4] Guarner J. (2020). Three Emerging Coronaviruses in Two Decades the Story of SARS, MERS, and Now COVID-19. Am. J. Clin. Pathol..

[5] Xiao C., Li X., Liu S., Gao S.J., Gao F. (2020). HIV-1 did not contribute to the 2019-nCoV genome. Emerg. Infect. Dis..

[6] Kandeel M., Al-Nazawi M. (2020). Virtual screening and repurposing of FDA approved drugs against COVID-19 main protease. Life Sci..

[7] Vellingiri B., Jayaramayya K., Iyer M., Kumar N.S., Subramaniam M.D. (2020). COVID-19: A promising cure for the global panic. Sci. Total Environ..

[8] Yan R., Zhang Y., Li Y., Xia L., Guo Y., Zhou Q. (2020). Structural basis for the recognition of SARS-CoV-2 by full-length human ACE2. Science.

[9] Kumari L.S., Mazumder A., Kumar V., Gupta S. (2019). Synthesis and biological potentials of quinoline analogues: A review of literature. Mini-Rev. Org. Chem..

[10] Zhang J., Wang S., Ba Y., Xu Z. (2019). 1,2,4-Triazole-quinoline/quinolone hybrids as potential anti-bacterial agents. Eur. J. Med. Chem..

[11] Al Matarneh C., Sardaru M., Apostu M., Rosca I., Ciobanu C., Mangalagiu I.I., Danac R. (2019). Synthesis and antibacterial evaluation of new pyrrolo[3’,4′:3,4]pyrrolo[1,2-a]quinoline and pyrrolo[3′,4′:3,4]pyrrolo[1,2-a]isoquinoline derivatives. Studia UBB Chem..

[12] Kalaria P.N., Karad S.C., Raval D.K. (2018). A review on diverse heterocyclic compounds as the privileged scaffolds in antimalarial drug discovery. Eur. J. Med. Chem..

[13] Ajani O.O., Iyaye K.T., Audu O.Y., Kuye A.O., Olanrewaju I.O. (2018). Microwave Assisted Synthesis and Antimicrobial Potential of Quinoline-Based 4-Hydrazide-Hydrazone Derivatives. J. Heterocycl. Chem..

[14] Hu Y.Q., Gao C., Zhang S., Xu L., Xu Z., Feng L.S., Wu X., Zhao F. (2017). Quinoline hybrids and their antiplasmodial and antimalarial activities. Eur. J. Med. Chem..

[15] Mantu D., Antoci V., Moldoveanu C., Zbancioc G., Mangalagiu I.I. (2016). Hybrid imidazole (benzimidazole)/pyridine (quinoline) derivatives and evaluation of their anticancer and antimycobacterial activity. J. Enz. Inhib. Med. Chem..

[16] Zhong F., Geng G., Chen B., Pan T., Li Q., Zhang H., Bai C. (2015). Identification of benzenesulfonamide quinoline derivatives as potent HIV-1 replication inhibitors targeting Rev protein. Org. Biomol. Chem..

[17] Sen C., Sahoo T., Singh H., Suresh E., Ghosh S.C. (2019). Visible light-promoted photocatalytic C-5 carboxylation of 8-aminoquinoline amides and sulfonamides via a single electron transfer pathway. J. Org. Chem..

[18] Pascual-Álvarez A., Topala T., Estevan F., Sanz F., Alzuet-Piña G. (2016). Photoinduced and self-activated nuclease activity of copper(II) complexes with *N*-(quinolin-8-yl)quinolin-8-sulfonamide-DNA and bovine serum albumin binding. Eur. J. Inorg. Chem..

[19] Meeusen J.W., Tomasiewicz H., Nowakowski A., Petering D.H. (2011). TSQ (6-methoxy-8-p-toluenesulfonamido-quinoline), a common fluorescent sensor for cellular zinc, images zinc proteins. Inorg. Chem..

[20] Rouffet M., De Oliveira C.A.F., Udi Y., Agrawal A., Sagi I., McCammon J.A., Cohen S.M. (2010). From sensors to silencers: Quinoline- and benzimidazole-sulfonamides as inhibitors for zinc proteases. J. Am. Chem. Soc..

[21] Macías B., García I., Villa M.V., Borrás J., Castineiras A., Sanz F. (2003). Synthesis and structural characterization of zinc complexes with sulfonamide containing 8-aminoquinoline. Z. Anorg. Allg. Chem..

[22] Fahrni C.J., O’Halloran T.V. (1999). Aqueous coordination chemistry of quinoline-based fluorescence probes for the biological chemistry of zinc. J. Am. Chem. Soc..

[23] Da Silva L.E., de Sousa P.T., Joussef A.C., Piovezan C., Neves A. (2008). Synthesis, structure and physicochemical properties of zinc and copper complexes based on sulfonamides containing 8-aminoquinoline ligands. Quim. Nova.

[24] Da Silva L.E., Joussef A.C., Pacheco L.K., Da Silva D.G., Steindel M., Rebelo R.A., Schmidt B. (2007). Synthesis and in vitro evaluation of leishmanicidal and trypanocidal activities of *N*-quinolin-8-yl arylsulfonamides. Bioorg. Med. Chem..

[25] Da Silva L.E., de Sousa P.T., Maciel E.N., Numes R.K., Eger I., Steindel M., Rebelo R.A. (2010). In vitro antiprotozoal evaluation of zinc and copper complexes based on sulfonamides containing 8-aminoquinoline ligands. Lett. Drug Des. Discov..

[26] Diaz J.R.A., Baldo M.F., Echeverría G., Baldoni H., Vullo D., Soria D.B., Supuran C.T., Cami G.E. (2016). A substituted sulfonamide and its Co (II), Cu (II), and Zn (II) complexes as potential antifungal agents. J. Enz. Inhib. Med. Chem..

[27] Antoci V., Cucu D., Zbancioc G., Moldoveanu C., Mangalagiu V., Amariucai-Mantu D., Aricu D., Mangalagiu I.I. (2020). Bis-(imidazole/benzimidazole)-pyridine derivatives: Synthesis, structure and antimycobacterial activity. Future Med. Chem..

[28] Olaru A.M., Vasilache V., Danac R., Mangalagiu I.I. (2017). Antimycobacterial activity of nitrogen heterocycles derivatives: 7-(pyridine-4-yl)-indolizine derivatives. Part VII. J. Enz. Inhib. Med. Chem..

[29] Mantu D., Antoci V., Nicolescu A., Delenu C., Vasilache V., Mangalagiu I.I. (2017). Synthesis, stereochemical studies and antimycobacterial activity of new acetyl-hydrazines pyridazinone. Curr. Org. Synth..

[30] Al Matarneh C.M., Shova S., Mangalagiu I.I., Danac R. (2016). Synthesis, structure, antimycobacterial and anticancer evaluation of new pyrrolo-(phenanthroline) derivatives. J. Enz. Inhib. Med. Chem..

[31] Aricu A., Ciocarlan A., Lungu L., Barba A., Shova S., Zbancioc G., Mangalagiu I.I., D’Ambrosio M., Vornicu N. (2016). Synthesis of new antibacterial and antifungal drimane sesquiterpenoids with azaheterocyclic units. Med. Chem. Res..

[32] Al Matarneh C., Ciobanu C.I., Mangalagiu I.I., Danac R. (2016). Design, synthesis and antimycobacterial activity of some new azaheterocycles: 4,7-phenanthroline with p-halogeno-benzoyl skeleton. Part VI. J. Serb. Chem. Soc..

[33] Danac R., Al Matarneh C.M., Shova S., Daniloaia T., Balan M., Mangalagiu I.I. (2015). New indolizines with phenanthroline skeleton: Synthesis, structure, antimycobacterial and anticancer evaluation. Bioorg. Med. Chem..

[34] Danac R., Daniloaia T., Antoci V., Vasilache V., Mangalagiu I.I. (2015). Design, synthesis and antimycobacterial activity of some new azaheterocycles: Phenanthroline with p-halo-benzoyl skeleton. Part, V. Lett. Drug Des. Discov..

[35] Balan A.M., Miron A., Tuchilus C., Rotinberg P., Mihai C.T., Mangalagiu I.I., Zbancioc G. (2014). Synthesis and in vitro analysis of novel dihydroxyacetophenone derivatives with antimicrobial and antitumor activities. Med. Chem..

[36] Kuchkova K., Aricu A., Barba A., Vlad P., Shova S., Secara E., Ungur N., Tuchilus C., Zbancioc G., Mangalagiu I.I. (2014). Design, syntheses and antimicrobial activity of some novel homodrimane sesquiterpenoids with diazine skeleton. Med. Chem. Res..

[37] Tucaliuc R., Cotea V., Niculaua M., Tuchilus C., Mantu D., Mangalagiu I.I. (2013). New pyridazine–fluorine derivatives: Synthesis, chemistry and biological activity. Part II. Eur. J. Med. Chem..

[38] Mantu D., Antoci V., Mangalagiu I.I. (2013). Design, synthesis and antituberculosis activity of some new pyridazine derivatives: Bis-pyridazine. Part IV. Infect. Disord. Drug Targets.

[39] Mantu D., Luca M.C., Moldoveanu C., Zbancioc G., Mangalagiu I.I. (2010). Synthesis and antituberculosis activity of some new pyridazine derivatives. Part II. Eur. J. Med. Chem..

[40] Balan A.M., Florea O., Moldoveanu C., Zbancioc G., Iurea D., Mangalagiu I.I. (2009). Diazinium salts with dihydroxyacetophenone skeleton: Syntheses and antimicrobial activity. Eur. J. Med. Chem..

[41] CLSI Document M07-A11 (2018). Methods for Dilution Antimicrobial Susceptibility Tests for Bacteria That Grow Aerobically; Approved Standard.

[42] Kavanagh A., Ramu S., Gong Y., Copper M.A., Blaskovich M.A.T. (2019). Effects of microplate type and broth additives on microdilution MIC susceptibility assays. Antimicrob. Agents Ch..

[43] Supuran C.T. (2008). Carbonic anhydrases: Novel therapeutic applications for inhibitors and activators. Nat. Rev. Drug Discov..

