# Supplementary material for: Hybrid Quinoline-Sulfonamide Complexes (M2+) Derivatives with Antimicrobial Activity
Source: Molecules. 2020 Jun 26;25(12):2946. doi: 10.3390/molecules25122946 (PMC7356327; doi:10.3390/molecules25122946)

Supporting information

Hybrid Quinoline-Sulfonamide Complexes (M^{2+}) Derivatives with Antimicrobial Activity

Dumitrela Diaconu [1,2], Violeta Mangalagiu [2,*], Dorina Amariucai-Mantu [1], Vasilichia Antoci [1], Cristian Levente Giuroiu [3,*] and Ionel I. Mangalagiu [1,2,*]

1 Faculty of Chemistry, Alexandru Ioan Cuza University of Iasi, 11 Carol Bvd, 700506 Iasi, Romania; cucu.dumitrela@yahoo.com (D.D.); dorinaiasi@yahoo.com (D.A.-M); vasilichia2004@yahoo.com (V.A.)

2 Institute of Interdisciplinary Research—CERNESIM Center, Alexandru Ioan Cuza University of Iasi, 11 Carol Bvd, 700506 Iasi, Romania

3 Endodontics, Faculty of Dental Medicine, Grigore T. Popa University of Medicine and Pharmacy, 16 Universităţii Str., 700115 Iasi, Romania

* Correspondence: violeta.mangalagiu@uaic.ro (V.M.); giuroiu.cristian@umfiasi.ro (C.L.G.); ionelm@uaic.ro (I.I.M.)

<u>^{1}H and ^{13}C{^{1}H}-NMR spectra</u>

4-chloro-*N*-(quinolin-8-yl)benzenesulfonamide (**3a**)

^{1}H-NMR (500 MHz, DMSO-d_6)

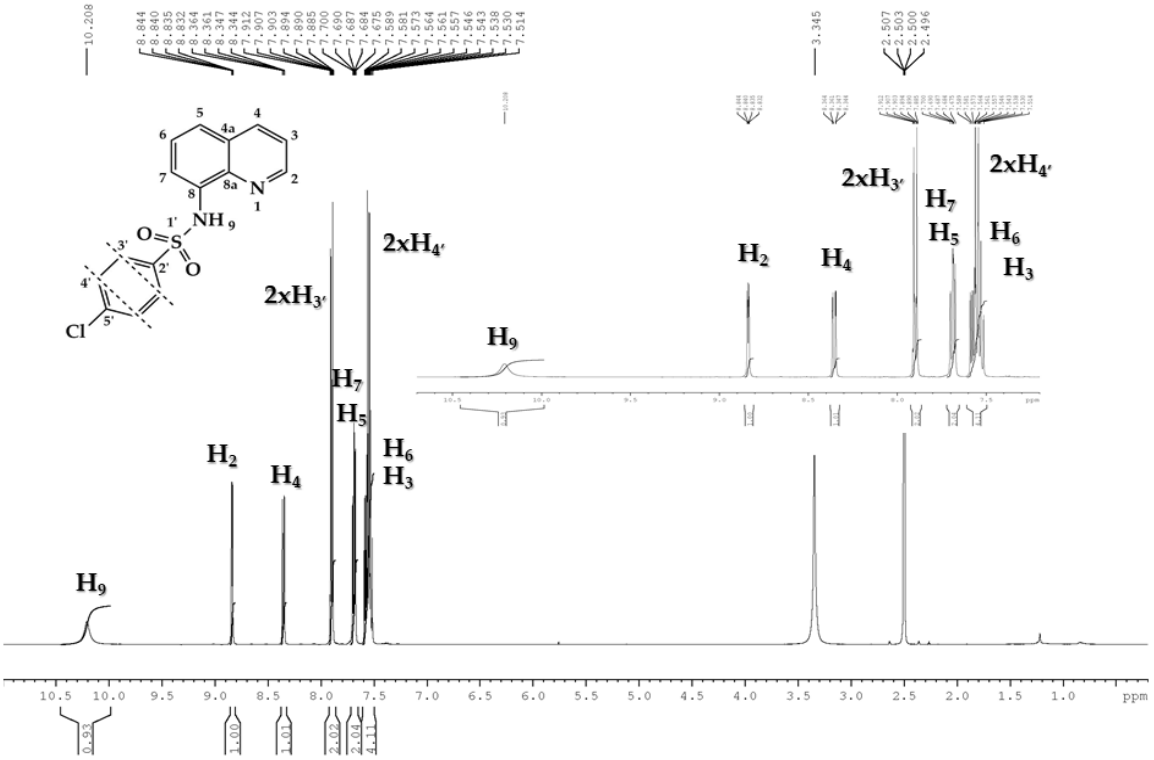

4-chloro-*N*-(quinolin-8-yl)benzenesulfonamide (**3a**)

$^{13}C\{^{1}H\}$-NMR (125 MHz, DMSO-*d*$_6$)

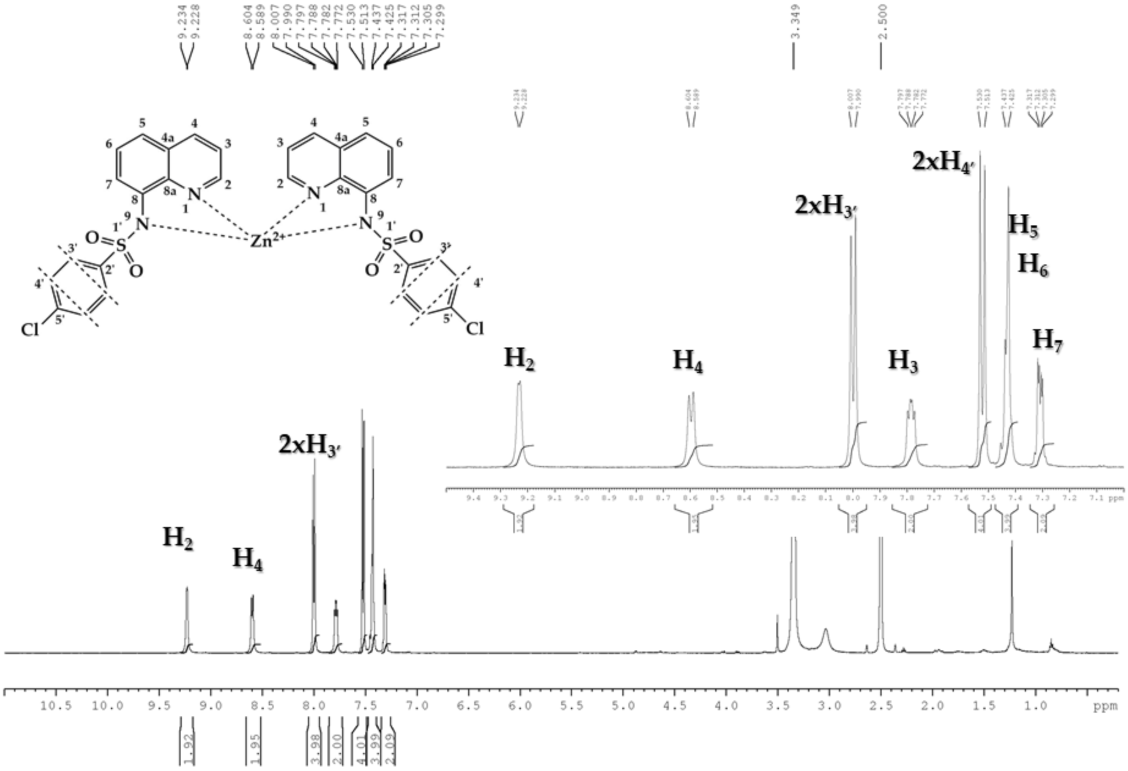

[Zn(*N*-(quinoline-8-yl)-4-chloro-benzenesulfonamide)$_2$] (**4a**)

^{1}H-NMR (500 MHz, DMSO-*d*$_6$)

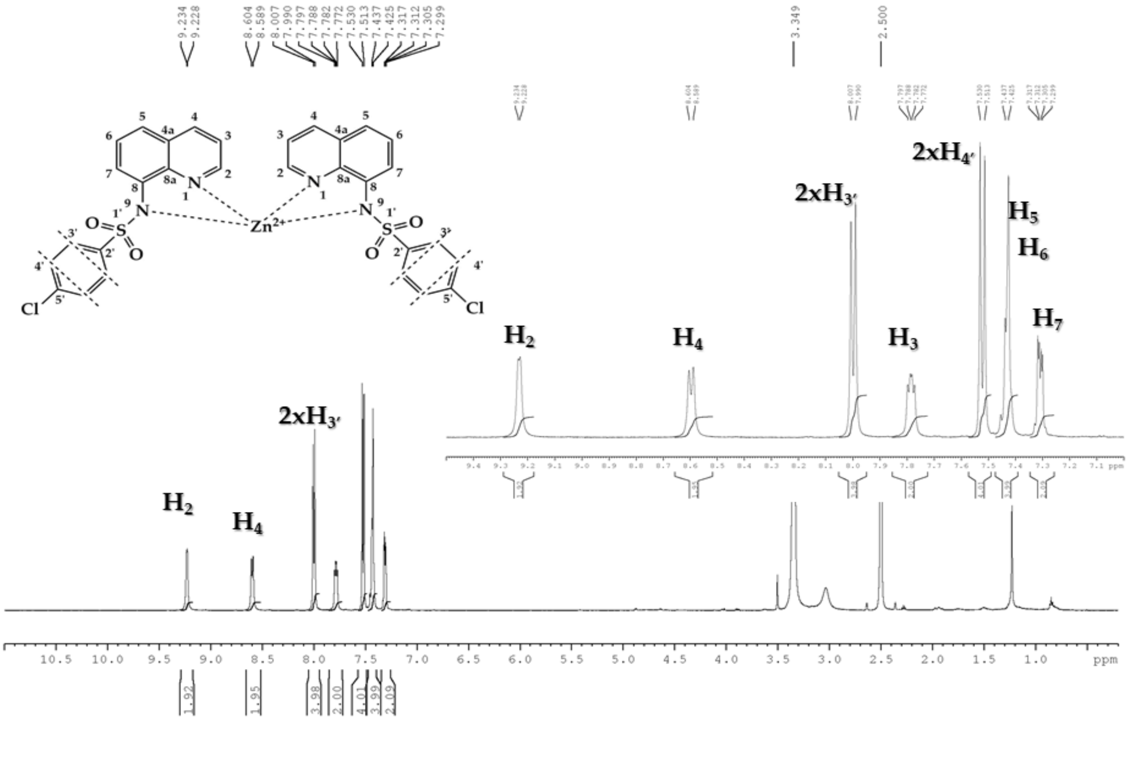

[Zn(*N*-(quinoline-8-yl)-4-chloro-benzenesulfonamide)$_2$] (**4a**)

$^{13}C\{^1H\}$-NMR (125 MHz, DMSO-*d*$_6$)

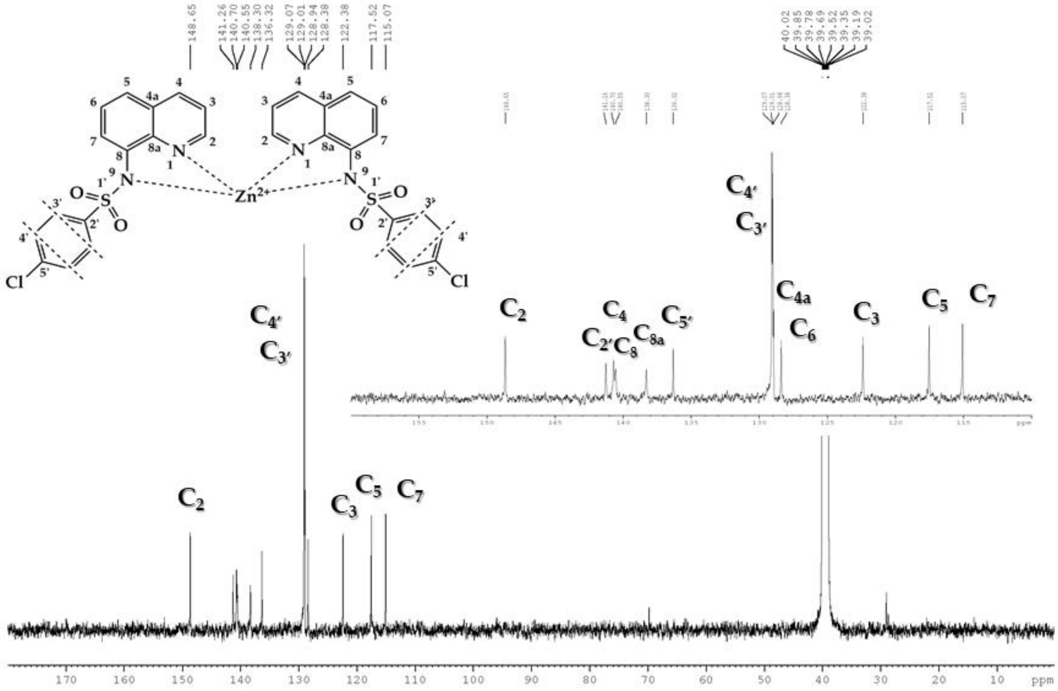

[Cd(*N*-(quinoline-8-yl)-4-chloro-benzenesulfonamide)$_2$] **(4d)**

^1H-NMR (500 MHz, DMSO-*d*$_6$)

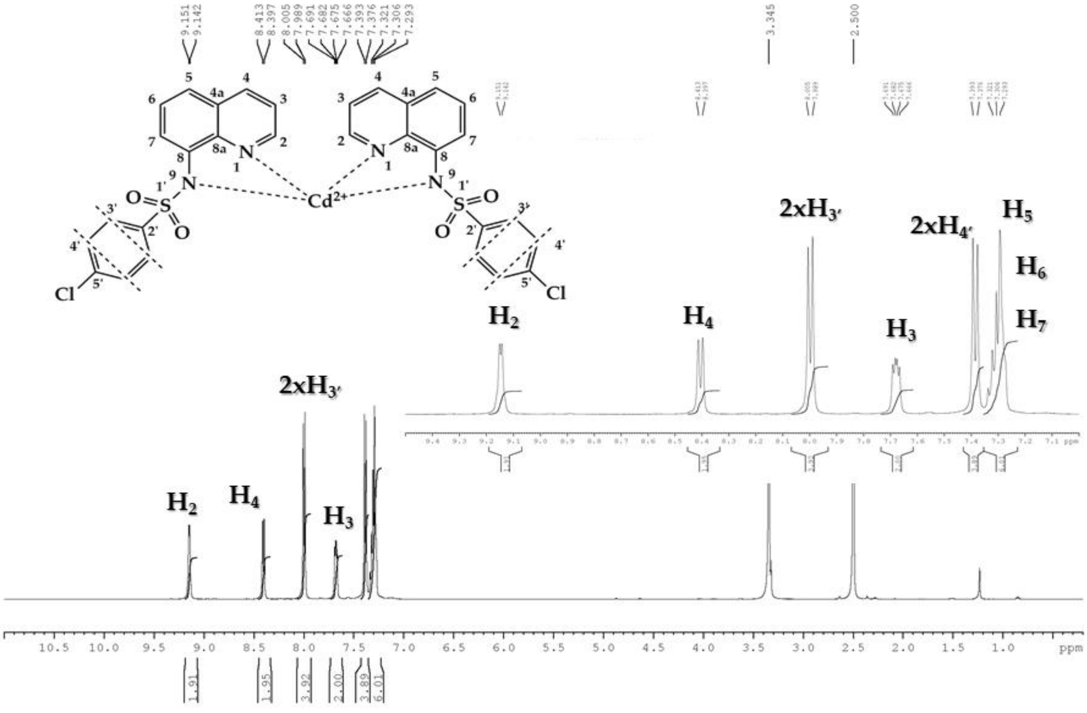

[Cd(*N*-(quinoline-8-yl)-4-chloro-benzenesulfonamide)$_2$] **(4d)**

$^{13}C\{^1H\}$-NMR (125 MHz, DMSO-d_6)

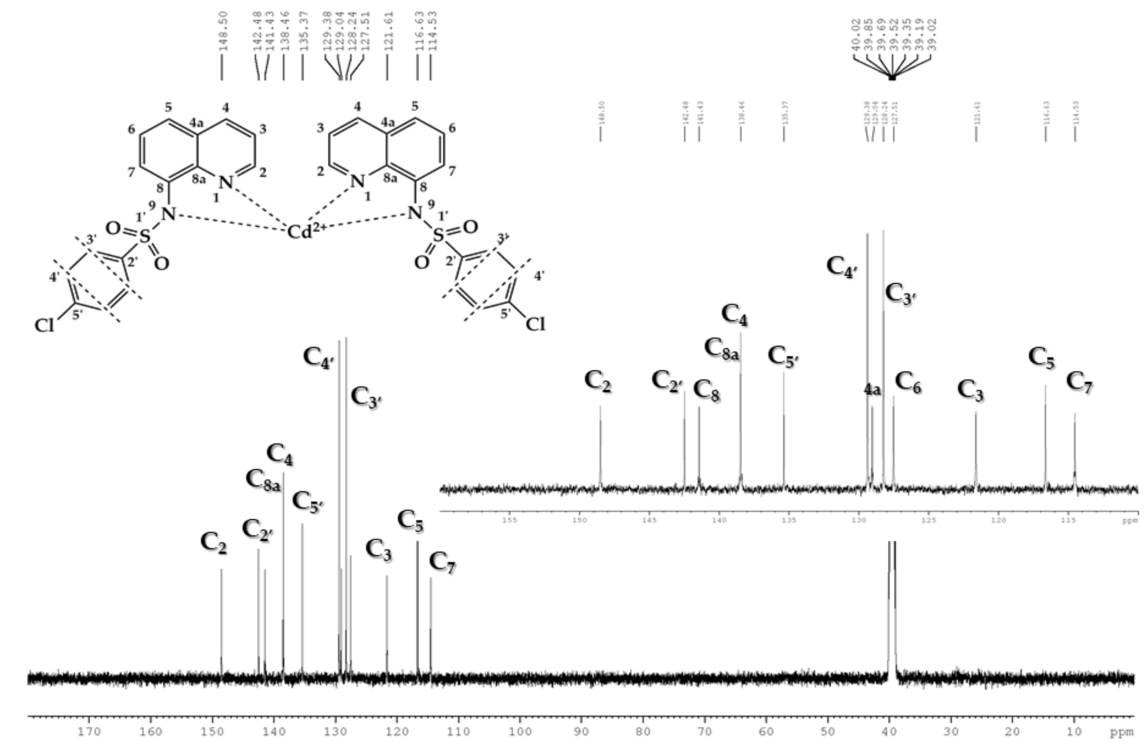

[Cd(*N*-(quinoline-8-yl)-4-chloro-benzenesulfonamide)$_2$] **(4d)**

2D-HMBC detail

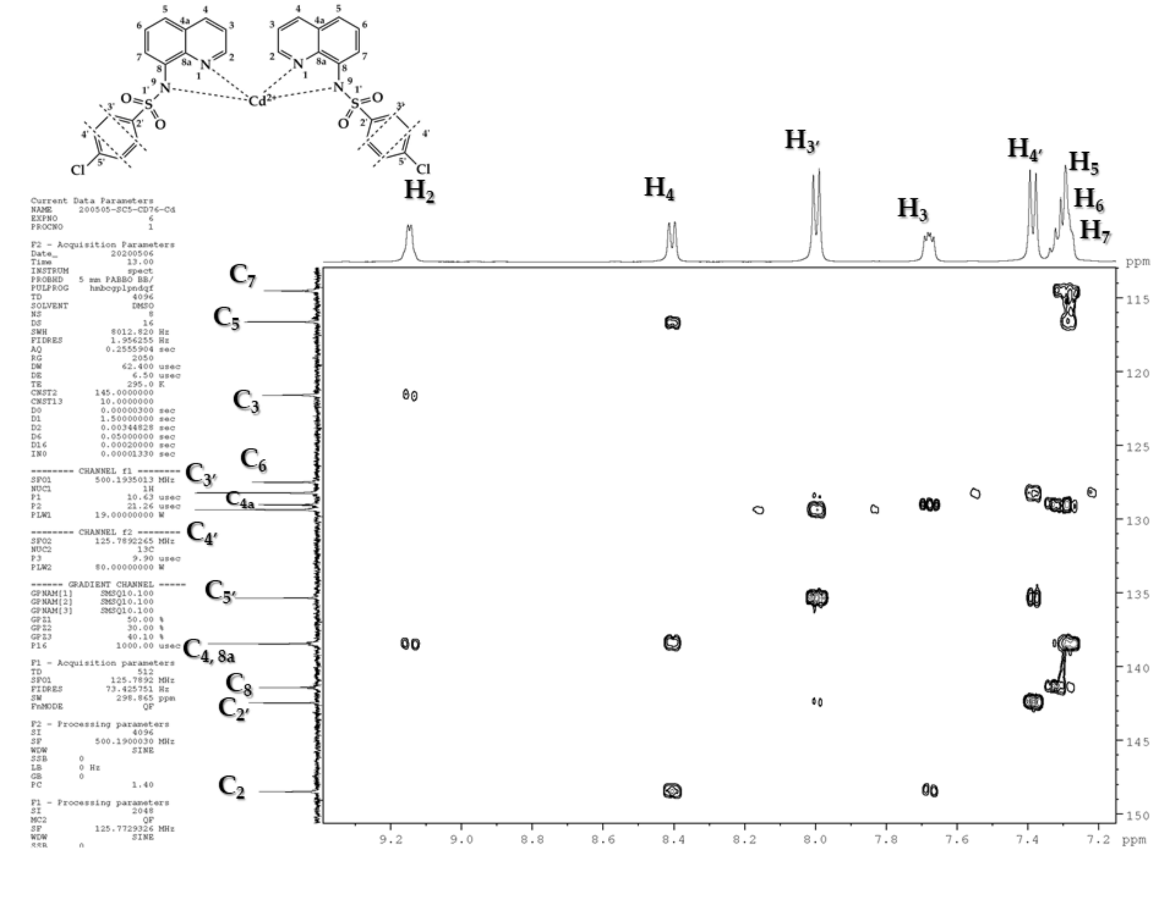

CheckCIF files for X-ray data

[Cu(*N*-(quinoline-8-yl)-4-chloro-benzenesulfonamide)$_2$] **(4b)**

checkCIF/PLATON report

Structure factors have been supplied for datablock(s) cd001tr_cryo

THIS REPORT IS FOR GUIDANCE ONLY. IF USED AS PART OF A REVIEW PROCEDURE FOR PUBLICATION, IT SHOULD NOT REPLACE THE EXPERTISE OF AN EXPERIENCED CRYSTALLOGRAPHIC REFEREE.

No syntax errors found. CIF dictionary Interpreting this report

Datablock: cd001tr_cryo

```
Bond precision:  C-C = 0.0030 A          Wavelength=1.54184

Cell:            a=17.1674(5)    b=15.2452(5)        c=24.2159(8)
                 alpha=90        beta=104.567(1)     gamma=90
Temperature:     100 K
```

	Calculated	Reported
Volume	6134.1(3)	6134.1(3)
Space group	I 2/a	I 2/a
Hall group	-I 2ya	-I 2ya
Moiety formula	4(C30 H20 Cl2 Cu N4 O4 S2), C H2 Cl, Cl	C30 H20 Cl2 Cu N4 O4 S2, 0.25(C H2 Cl2)
Sum formula	C121 H82 Cl10 Cu4 N16 O16 S8	C30.25 H20.50 Cl2.50 Cu N4 O4 S2
Mr	2881.21	720.29
Dx,g cm-3	1.560	1.560
Z	2	8
Mu (mm-1)	4.650	1.050
F000	2924.0	2924.0
F000'	2927.51	
h,k,lmax	21,18,29	20,18,29
Nref	5932	5816
Tmin,Tmax	0.801,0.836	0.613,1.000
Tmin'	0.656	

```
Correction method= # Reported T Limits: Tmin=0.613 Tmax=1.000
AbsCorr = MULTI-SCAN

Data completeness= 0.980          Theta(max)= 71.024

R(reflections)= 0.0367( 5669)     wR2(reflections)= 0.1009( 5816)

S = 1.066                  Npar= 416
```

The following ALERTS were generated. Each ALERT has the format
test-name_ALERT_alert-type_alert-level.
Click on the hyperlinks for more details of the test.

Alert level A

```
ABSMU01_ALERT_1_A  The ratio of given/expected absorption coefficient lies
               outside the range 0.90 <> 1.10
            Calculated value of mu =     4.650
            Value of mu given      =     1.050
PLAT051_ALERT_1_A Mu(calc) and Mu(CIF) Ratio Differs from 1.0 by .     342.81 %
```

Alert level C

```
PLAT031_ALERT_4_C Refined Extinction Parameter Within Range ......      2.667 Sigma
PLAT094_ALERT_2_C Ratio of Maximum / Minimum Residual Density ....       2.23 Report
PLAT336_ALERT_2_C Long Bond Distance for ..... C2        -Cl1           1.909 Ang.
PLAT911_ALERT_3_C Missing FCF Refl Between Thmin & STh/L=    0.600          6 Report
PLAT975_ALERT_2_C Check Calcd Resid. Dens.  0.56A   From C2              1.06 eA-3
```

Alert level G

```
PLAT003_ALERT_2_G Number of Uiso or Uij Restrained non-H Atoms ...          3 Report
PLAT042_ALERT_1_G Calc. and Reported MoietyFormula Strings  Differ     Please Check
PLAT045_ALERT_1_G Calculated and Reported Z Differ by a Factor ...       0.25 Check
PLAT083_ALERT_2_G SHELXL Second Parameter in WGHT  Unusually Large      13.98 Why ?
PLAT186_ALERT_4_G The CIF-Embedded .res File Contains ISOR Records          2 Report
PLAT300_ALERT_4_G Atom Site Occupancy of Cl1        Constrained at       0.25 Check
PLAT300_ALERT_4_G Atom Site Occupancy of C2         Constrained at       0.25 Check
PLAT300_ALERT_4_G Atom Site Occupancy of H2A        Constrained at       0.25 Check
PLAT300_ALERT_4_G Atom Site Occupancy of H2B        Constrained at       0.25 Check
PLAT300_ALERT_4_G Atom Site Occupancy of Cl2        Constrained at       0.25 Check
PLAT302_ALERT_4_G Anion/Solvent/Minor-Residue Disorder (Resd  2  )       100% Note
PLAT302_ALERT_4_G Anion/Solvent/Minor-Residue Disorder (Resd  3  )       100% Note
PLAT304_ALERT_4_G Non-Integer Number of Atoms in ..... (Resd  3  )       0.25 Check
PLAT432_ALERT_2_G Short Inter X...Y Contact  Cl2       ..C2              2.10 Ang.
                                                  x,y,z  =      1_555 Check
PLAT432_ALERT_2_G Short Inter X...Y Contact  Cl2       ..C2              2.68 Ang.
                                            3/2-x,y,1-z  =      2_656 Check
PLAT432_ALERT_2_G Short Inter X...Y Contact  O009      ..C2              2.99 Ang.
                                            3/2-x,y,1-z  =      2_656 Check
PLAT432_ALERT_2_G Short Inter X...Y Contact  C2        ..C2              2.70 Ang.
                                            3/2-x,y,1-z  =      2_656 Check
PLAT720_ALERT_4_G Number of Unusual/Non-Standard Labels .........          63 Note
PLAT789_ALERT_4_G Atoms with Negative _atom_site_disorder_group  #          1 Check
PLAT793_ALERT_4_G Model has Chirality at S002         (Centro SPGR)         R Verify
PLAT860_ALERT_3_G Number of Least-Squares Restraints ............          18 Note
PLAT912_ALERT_4_G Missing # of FCF Reflections Above STh/L=  0.600        109 Note
PLAT941_ALERT_3_G Average HKL Measurement Multiplicity ..........         3.0 Low
PLAT978_ALERT_2_G Number C-C Bonds with Positive Residual Density.          7 Info
PLAT992_ALERT_5_G Repd & Actual _reflns_number_gt Values Differ by          1 Check
```

---

2 **ALERT level A** = Most likely a serious problem - resolve or explain
0 **ALERT level B** = A potentially serious problem, consider carefully
5 **ALERT level C** = Check. Ensure it is not caused by an omission or oversight
25 **ALERT level G** = General information/check it is not something unexpected

4 ALERT type 1 CIF construction/syntax error, inconsistent or missing data

```
10 ALERT type 2 Indicator that the structure model may be wrong or deficient
 3 ALERT type 3 Indicator that the structure quality may be low
14 ALERT type 4 Improvement, methodology, query or suggestion
 1 ALERT type 5 Informative message, check
```

It is advisable to attempt to resolve as many as possible of the alerts in all categories. Often the minor alerts point to easily fixed oversights, errors and omissions in your CIF or refinement strategy, so attention to these fine details can be worthwhile. In order to resolve some of the more serious problems it may be necessary to carry out additional measurements or structure refinements. However, the purpose of your study may justify the reported deviations and the more serious of these should normally be commented upon in the discussion or experimental section of a paper or in the "special_details" fields of the CIF. checkCIF was carefully designed to identify outliers and unusual parameters, but every test has its limitations and alerts that are not important in a particular case may appear. Conversely, the absence of alerts does not guarantee there are no aspects of the results needing attention. It is up to the individual to critically assess their own results and, if necessary, seek expert advice.

Publication of your CIF in IUCr journals

A basic structural check has been run on your CIF. These basic checks will be run on all CIFs submitted for publication in IUCr journals (*Acta Crystallographica*, *Journal of Applied Crystallography*, *Journal of Synchrotron Radiation*); however, if you intend to submit to *Acta Crystallographica Section C* or *E* or *IUCrData*, you should make sure that full publication checks are run on the final version of your CIF prior to submission.

Publication of your CIF in other journals

Please refer to the *Notes for Authors* of the relevant journal for any special instructions relating to CIF submission.

PLATON version of 16/04/2020; check.def file version of 09/03/2020

Datablock cd001tr_cryo - ellipsoid plot

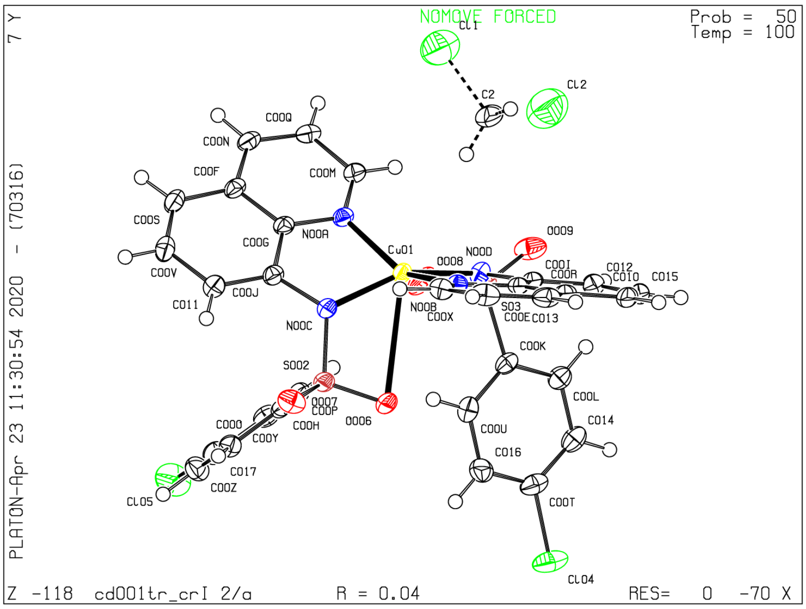

[Co(*N*-(quinoline-8-yl)-4-chloro-benzenesulfonamide)$_2$] (**4c**)

checkCIF/PLATON report

Structure factors have been supplied for datablock(s) cd002tr_cryo

THIS REPORT IS FOR GUIDANCE ONLY. IF USED AS PART OF A REVIEW PROCEDURE FOR PUBLICATION, IT SHOULD NOT REPLACE THE EXPERTISE OF AN EXPERIENCED CRYSTALLOGRAPHIC REFEREE.

No syntax errors found. CIF dictionary Interpreting this report

Datablock: cd002tr_cryo

```
Bond precision:  C-C = 0.0024 A          Wavelength=1.54184

Cell:           a=12.1381(2)    b=15.5893(2)        c=16.1980(3)
                alpha=90        beta=106.347(2)     gamma=90
Temperature:    100 K

                Calculated                   Reported
Volume          2941.15(9)                   2941.15(9)
Space group     P 21/n                       P 21/n
Hall group      -P 2yn                       -P 2yn
Moiety formula  C30 H20 Cl2 Co N4 O4 S2      ?
Sum formula     C30 H20 Cl2 Co N4 O4 S2      C30 H20 Cl2 Co N4 O4 S2
Mr              694.45                       694.45
Dx,g cm-3       1.568                        1.568
Z               4                            4
Mu (mm-1)       7.948                        7.958
F000            1412.0                       1412.0
F000'           1411.58
h,k,lmax        14,19,19                     14,19,19
Nref            5660                         5608
Tmin,Tmax       0.518,0.651                  0.235,1.000
Tmin'           0.023

Correction method= # Reported T Limits: Tmin=0.235 Tmax=1.000
AbsCorr = GAUSSIAN

Data completeness= 0.991          Theta(max)= 70.836

R(reflections)= 0.0259( 5425)     wR2(reflections)= 0.0693( 5608)

S = 1.037                  Npar= 388
```

The following ALERTS were generated. Each ALERT has the format
test-name_ALERT_alert-type_alert-level.
Click on the hyperlinks for more details of the test.

Alert level G

```
PLAT434_ALERT_2_G Short Inter HL..HL Contact Cl1      ..Cl1             3.35 Ang.
                                      -x,2-y,2-z  =      3_577 Check
PLAT794_ALERT_5_G Tentative Bond Valency for Co1       (II)      .       1.86 Info
PLAT912_ALERT_4_G Missing # of FCF Reflections Above STh/L=  0.600         51 Note
PLAT978_ALERT_2_G Number C-C Bonds with Positive Residual Density.         14 Info
```

```
  0 ALERT level A = Most likely a serious problem - resolve or explain
  0 ALERT level B = A potentially serious problem, consider carefully
  0 ALERT level C = Check. Ensure it is not caused by an omission or oversight
  4 ALERT level G = General information/check it is not something unexpected

  0 ALERT type 1 CIF construction/syntax error, inconsistent or missing data
  2 ALERT type 2 Indicator that the structure model may be wrong or deficient
  0 ALERT type 3 Indicator that the structure quality may be low
  1 ALERT type 4 Improvement, methodology, query or suggestion
  1 ALERT type 5 Informative message, check
```

It is advisable to attempt to resolve as many as possible of the alerts in all categories. Often the minor alerts point to easily fixed oversights, errors and omissions in your CIF or refinement strategy, so attention to these fine details can be worthwhile. In order to resolve some of the more serious problems it may be necessary to carry out additional measurements or structure refinements. However, the purpose of your study may justify the reported deviations and the more serious of these should normally be commented upon in the discussion or experimental section of a paper or in the "special_details" fields of the CIF. checkCIF was carefully designed to identify outliers and unusual parameters, but every test has its limitations and alerts that are not important in a particular case may appear. Conversely, the absence of alerts does not guarantee there are no aspects of the results needing attention. It is up to the individual to critically assess their own results and, if necessary, seek expert advice.

Publication of your CIF in IUCr journals

A basic structural check has been run on your CIF. These basic checks will be run on all CIFs submitted for publication in IUCr journals (*Acta Crystallographica*, *Journal of Applied Crystallography*, *Journal of Synchrotron Radiation*); however, if you intend to submit to *Acta Crystallographica Section C* or *E* or *IUCrData*, you should make sure that full publication checks are run on the final version of your CIF prior to submission.

Publication of your CIF in other journals

Please refer to the *Notes for Authors* of the relevant journal for any special instructions relating to CIF submission.

PLATON version of 22/12/2019; check.def file version of 13/12/2019

Datablock cd002tr_cryo - ellipsoid plot

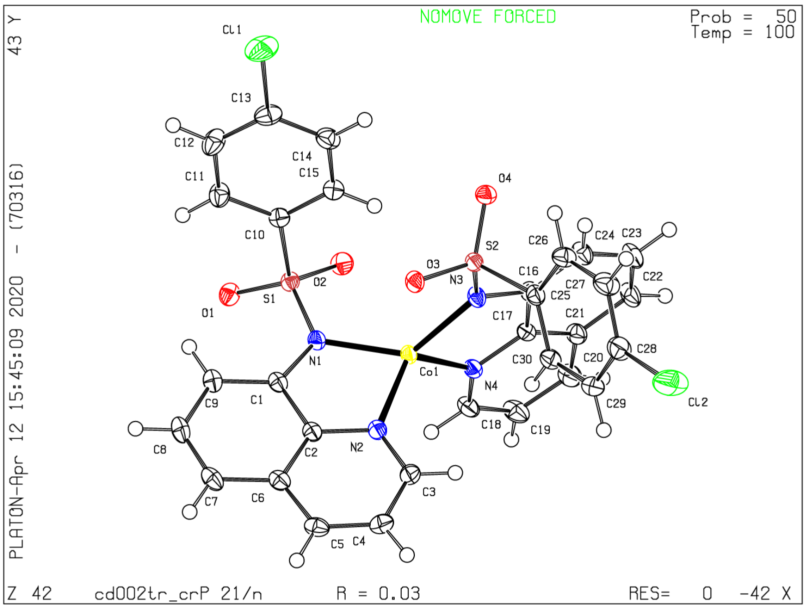

Supplement: Supplementary file 1 [file molecules-25-02946-s001.pdf]
